# Removal of heavy metals from dredging marine sediments via electrokinetic hexagonal system: A pilot study in Italy

**DOI:** 10.1016/j.heliyon.2024.e27616

**Published:** 2024-03-13

**Authors:** Erika Pasciucco, Francesco Pasciucco, Alessio Castagnoli, Renato Iannelli, Isabella Pecorini

**Affiliations:** Department of Energy, Systems Territory and Construction Engineering, Via C.F. Gabba 22, Tuscany, University of Pisa, Pisa, 56122, Italy

**Keywords:** Electrokinetic, Marine sediment decontamination, Hexagonal configuration, Metal removal

## Abstract

Among the several treatment options, electrokinetic (EK) remediation is recognized as an effective technique for the removal of heavy metals from low-permeability porous matrices. However, most of the EK decontamination research reported was performed on linear configuration systems at a laboratory scale. In this study, a series of experiments were performed on a pilot-scale system where the electrodes were arranged in a hexagonal configuration, to assess the improvement of the EK process in the removal of inorganic contaminants from sediments dredged in the harbor of Piombino, Italy. HNO_3_ was used as acid conditioning and both pH effect and treatment duration time were investigated. Sediment characterization and metal fractionation were also presented, in order to understand how the bioavailability of metals affects the process efficiency. The increase in pH due to the buffering capacity of the sediment in the sections close to the cathode favored the precipitation and accumulation of metals. However, the results highlighted that longer treatment times, combined with an efficient pH reduction, can improve treatment performance, resulting in high removal efficiencies for all the target metals considered (a percentage removal greater than 50% was reached for Cd, Ni, Pb, Cu and Zn). Compared to different EK configuration systems, the hexagonal configuration arrangement applied in our study provides better results for the remediation of dredged marine sediment.

## Highlights

• Pilot-scale hexagonal set-up improves electrokinetic removal of heavy metals in dredged marine sediments.
• A removal efficiency above 50% has been achieved for Cd, Ni, Pb, Cu and Zn.
• Longer treatment times improved decontamination efficiency.
• Better performance of the hexagonal configuration compared to conventional linear configuration systems.

## 1 Introduction

Sediment dredging for waterway maintenance poses an environmental issue in sediment management [1,2]. The main concern is related to the high pollution load introduced during ship transport and industrial activities. In particular, the contamination of harbor sediments by heavy metals and their complexation with organic matter represent crucial bottlenecks, as their high stability hinders their removal [3].

Traditional soil management strategies include environmentally unsustainable alternatives such as landfills or near-shore disposal facilities [4]. Nowadays, process sustainability [5,6] and resource recovery [7] represents a new paradigm for industrial activities [8], but the current technological readiness level (TRL) of some processes is still low [9].

In view of that, the development of new technologies for soil remediation in a circular economy perspective is one of the main environmental challenges. The efficiency of process is closely related to soil characteristics, such as pollutant concentration, organic matter content, particle size distribution, and alkalinity; therefore, based on matrix complexity, specific treatments may be required [10].

Focusing on marine sediments, the high salinity, low hydraulic permeability and high content of carbonates and organic matter often make common treatment technologies (e.g pump-and-treat or soil washing) inadequate or inefficient [11]. Among the available treatment options, electrokinetic (EK) decontamination is recognized as an effective technique for the removal of heavy metals from low-permeability porous matrices [12]. In addition, its feasible implementation both in situ and ex-situ and coupling with other remediation technologies, such as phytoremediation and Fenton processes, represent further advantages [13,14].

EK technology exploits the application of an electric field to promote the mobilization of metal ions towards the electrodes through three main transport mechanisms: electroosmosis, electromigration and electrophoresis [15,16].

Furthermore, the applied electric field promotes the water electrolysis with the production of H^+^ and OH^−^ ions, which generate a charge gradient along the solid matrix due to the transport phenomena described above, making it essential to adjust the pH to avoid undesirable effects that can inhibit the transport mechanisms (e.g precipitation of hydroxides and carbonates) [17].

Nevertheless, the electric field active area is the main driving force of the process, and the efficiency of the system is closely related to the configuration of the electrodes [18]. Regarding inorganic contaminants, over the years, various electrode configurations have been studied to increase the removal efficiency [19]. Compared to a 1-D configuration, a 2-D configuration achieves greater decontamination by reducing specific electricity consumption [20]. In this context, Kim et al. [21] proved that a pilot-scale 2-D hexagonal configuration applied to agricultural saline lands shows high salt removal efficiencies; however, most of the studies focused on heavy metal remediation from soils [22,23]. Therefore, the different behaviour of the sediments due to the high salinity and carbonate content must be taken into account.

In a recent study, the performance of a one-dimensional configuration was compared with that of a two-dimensional configuration in terms of pollutant removal efficiency and electric energy consumption. The 2-D hexagonal configuration showed better performance and allowed an increase in the removal efficiency of Cu and Pb by more than 80% [24]. Consistently, another in situ EK study demonstrated that the hexagonal configuration can be a valid option for the remediation of paddy rice fields polluted with As, Pb, and Cu [25].

At the same time, many authors have addressed this topic, focusing on sediment remediation.

Ammami et al. [26] performed bench-scale EK tests to evaluate the effects of various operation settings for a single-stage treatment aimed at removing heavy metals and PAHs from dredged silt. Citric acid, a chelating agent, and various surfactants have been combined. The tests showed satisfying results (50%, 30%, and 35% of Zn, Pb, and Cd were removed, respectively) using a dosage of 0.1 M of citric acid.

A similar study on the extraction of heavy metals from marine sediments tested various processing fluids such as EDTA, citric acid, HNO_3_ and HCl, where HCl has been shown to be the best agent for the extraction of Ni, Cu, Zn, and Pb [3].

To the best of our knowledge, a 2-D hexagonal configuration plant for harbor sediment remediation has not yet been optimized. In this study, a particular two-dimensional hexagonal EK configuration at pilot-scale was applied for the first time for marine sediment remediation, in order to evaluate the heavy metal removal efficiency of EK technology.

## 2 Materials and method

### 2.1 Sediment sampling and characterization

Sediment samples were dredged within the Darsena Pescherecci area in the harbor of Piombino (Italy), which is identified by the P84 code in Fig. S1a. Sampling was performed by divers using a Van Veen grab sampler with a sampling area of 250 cm^2^ and a total volume of 3.14 L, from the surface layer of the seafloor at a water depth of about 7 m. A total volume of 0.3 m^3^ of sediments were collected. After dredging, samples were homogenized by mixing for 30 min with a mechanical stirrer and stored in a refrigerator at 4 °C before the EK experiment was carried out. The granulometric analysis was conducted by the Italian Institute for Environmental Protection and Research (ISPRA), analysing sediment layers at different depths of the seabed surface (Fig. S1b). The sample at 0–10 cm depth was representative of the superficial layer; whereas, the sample at 30–50 cm was representative of the deep one. Sediment samples were fine-grained both at 0–10 and 30–50 cm depth, and the silt component (about 70%) was the predominant one. In Table 1, the chemical characterization of sediment samples was reported, showing threshold concentrations of each parameter according to the Italian Legislative Decree 152/2006 for polluted soils and subsoils [27]. Heavy metal concentration was measured by ICP-OES, and the limit of detection was reported for each parameter. All tests were performed on two replicates. For each replicate, a number of three readings were performed, so the heavy metal concentration was calculated based on the average of the readings.

**Table 1:** Characterization of P84 dredged sediment.

Parameter	Unit	Value	Limit concentration^a^	Limit of detection
pH	–	8.48 ± 0.25	–	–
Humidity	% m/m	43.1 ± 2.6	–	–
Cd	mg/kg ss	4.4 ± 0.7	2	0.5
Cr	mg/kg ss	169.0 ± 36.0	150	0.20
Ni	mg/kg ss	91.7 ± 10.8	120	1.0
Pb	mg/kg ss	478.0 ± 60.0	100	2.0
Cu	mg/kg ss	180.0 ± 23.0	120	2.0
Zn	mg/kg ss	2006.0 ± 244.0	150	1.0
Total organic matter	% m/m ss	8.60 ± 1.4	–	–

a Limit concentration established according to the Italian Legislation Decree 152/2006.

### 2.2 Experimental set-up

A schematic representation of the pilot-scale hexagonal configuration system was shown in Fig. S2a, consisting of a hollow cylinder 10 cm thick and 46 cm high, with a central chamber filled with 60 kg of sediment to be treated. A diagram of all components of the pilot-scale experimental setup and their dimensions were shown in Figs. S2b and S2c, respectively.

The electrodes were arranged in a hexagonal configuration: a cathode was placed in the middle and six anodes at the corners of the polygon. The cathode was 20 cm away from each anode, as well as the distance between the anodes was 20 cm. Both anodes and cathode were made of titanium mesh with a Mixed Metal Oxide (MMO) coating supplied by Industrie de Nora S. p.a (Italy) and were inserted into the respective electrode compartments filled with tap water. Sample P84 was collected from the site and subsequently homogenized before being placed in the central chamber for the experiment.

In order to prevent material transport and rapid outflow of electrolyte solutions, the solid matrix was isolated from the electrode compartments via a metal mesh coupled with filter paper.

The anolyte solution was recirculated by a magnetic drive pump. The levels of the anolyte and catholyte solutions were kept constant and connected to two tanks of 2 L each.

According to Iannelli et al. [4], among the various conditioning agents, HNO_3_ was the best compromise for reducing the pH of the sediments. For this reason, in this study the pH of the catholyte solution was kept constant (pH = 3) by the addition of HNO_3_ (4.5 M) and constantly monitored using a pH meter. The total amount of nitric acid added was approximately 70 L. The electrokinetic process was carried out for a duration of 70 and 95 days using a DC power generator EA-PSI 9080-60 DT 1500W. Preliminary tests showed that currents higher than 5 A induced sediment heating, involving the inhibition of the electrokinetic process.

Thus, a current value of 5 A was considered optimal for establishing the electric field without side effects and was kept constant throughout the test. As a result, the voltage changed as a function of the current intensity set. The voltage trend over time at a fixed current of 5 A is shown Fig. S3.

### 2.3 Analytical method

The trend of the electroosmotic flow (EOF) was determined by measuring the difference in volume in the electrode reservoirs through mass balance.

The resistivity was calculated according to the following equation:

(1) $$\rho =\frac{2\pi rh}{l}\times \frac{\Delta V}{I}$$

where 2πrh is the lateral surface of cylinder (m^2^) with high (h) equal to 0.46 m and radius (r) equal to 0.15 m, *l* is the width of the matrix (m), ΔV is the average voltage (V) and I the electrical current (A), respectively.

Metal speciation in the sediment was determined through sequential extraction, according to the procedure recommended by the Standards, Measurements and Testing (former BCR) Program of the European Commission. The BCR procedure is a standardized procedure for which there is a certified reference material (BCR-701), which allows validation of analytical performance [28] It consisted of metal extraction into three fractions: soluble and exchangeable, reducible, and oxidable. The residual fraction represents the mineral's non-extractable form. The details of the reagents used for extraction were reported in Table S2. The concentration of heavy metals in each extracted fraction and after EK treatment was determined by ICP-OES (limit of detection was reported in Table 1).

The removal efficiency was calculated by Eq. (1):

(2) $$Removal\;efficiency\;\left(\%\right)=\left(\frac{C_{0}-C_{f}}{C_{0}}\right)\times 100$$

where C_0_ and C_f_ are the initial concentration at time 0 and the final concentration at time t, after 70 and 95 days. The electric energy consumption (EEC) was calculated by the following equation (Eq. (2)):

(3) EEC(kWh)=[(I×V×A)×Δt]

where *I* (A/m^2^*)* and *V* (V) are the current and voltage applied, respectively, *A* (m^2^) is the treated sediment area and Δt (h) is the duration time of the tests. Since the voltage changes as a function of the current (which is kept constant), an average value was considered.

## 3 Results and discussion

### 3.1 Heavy metal fractionation

**Fig. 1:**
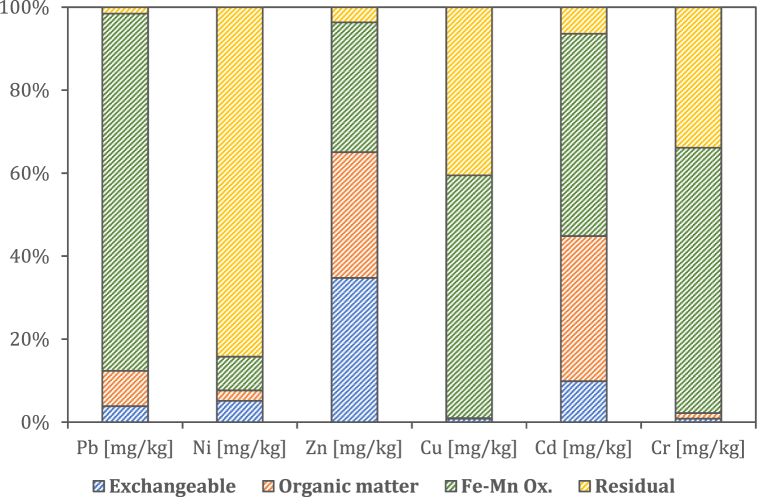
Heavy metal fractionation.

To evaluate heavy metal bioavailability, the sequential extraction was performed on five replicates of P84 sample. The results are shown in Fig. 1. As it can be observed, most of metals split into the Fe-Mn oxide fraction, which is particularly high for Pb (about 86%), Cr (64%) and Cu (about 60%). Contrary, in the case of Ni, the fraction bound to the Fe-Mn oxides does not exceed 10%; however, it is particularly high in the residual fraction (greater than 80%). With the exception of Ni, the residual fraction is less than 10% for Zn, Cd, and Pb, while it is higher for Cu (about 40%) and Cr (about 34%). The fraction bound to organic matter is particularly significant only for Cd (34%) and Zn (26%). The exchangeable fraction is negligible for all metals (below 10%), except Zn (35%) and Cd (10%). A significant Ni content as a residual fraction could make the removal of this metal more difficult. Conversely, elements such as Pb, which split predominantly into the fraction bound to the Fe-Mn oxides, can be more easily removed by reduction. However, it must be considered that a low contribution of the mobile fraction made the decontamination of sediment less efficient.

### 3.2 Trend of the electroosmotic flow (EOF)

**Fig. 2:**
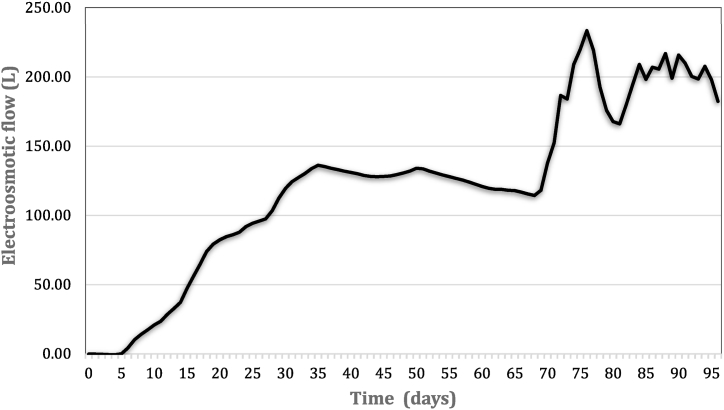
Electroosmotic flow.

Fig. 2 shows the variation of the EOF (cumulative one) with elapsed time. The electroosmotic flow is closely related to the zeta potential of sediment particles, which is negative when the pH is higher than the pH at the point of zero charge (pHpzc). In this case, the diffuse double layer is positively charged, and the electroosmotic flow moves towards the cathode. Conversely, when pH < pHpzc the zeta potential changed from a negative to a positive value, resulting in a reversal of the electroosmotic flow towards the anode. In our study, the direction of the electroosmotic flow occurred towards the cathode up to 33 days of treatment. The increase in electroosmotic flow can be explained by a high buffer capacity of the sediment and a low pH reduction efficiency along the matrix. Interestingly, the increase in electroosmotic flow corresponded to an increase in resistivity and voltage at the beginning of the experiment. This was related to the gradual removal of chlorides within dredged sediment, resulting in the development of chlorine gas in the anodic compartment. However, as can be seen in Fig. 2, the electroosmotic flow decreased after 51 days of treatment and stayed moderately negative until day 71. The reversal of the electroosmotic flow was attributed to the pH reduction by addition of nitric acid: the zeta potential changed (pH < pH_pzc_) and the electroosmotic flow occurred towards the anode. In addition to the pH reduction, acidification with HNO_3_ affected the pore solution, since introduced an excess of NO_3_^−^ ions attracted towards the anode. The combination of these factors made the zeta potential positive, resulting in a positively charged surface that contributed to the decrease in the electroosmotic permeability coefficient. A similar trend was observed in the last 25 days of treatment, where the electroosmotic flow first occurred towards the cathode and then reversed towards the anode based on the considerations above.

### 3.3 Heavy metal removal

**Fig. 3:**
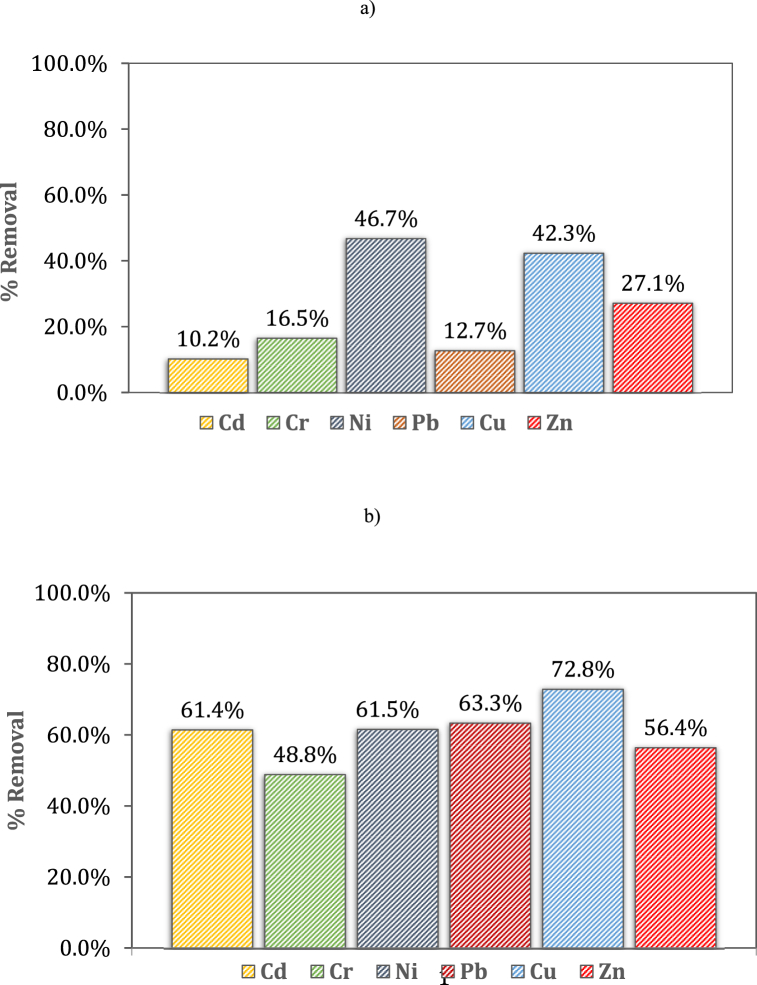
Heavy metal removal after 70 days (a) and 95 days (b).

The efficiency of heavy metals removal was monitored after 70 and 95 days, respectively. The results are shown in Fig. 3a and b. The initial concentration is uncertain due to the high inhomogeneity of the sample. For this reason, the removal percentage was calculated considering an estimate of the input value based on the mass balance of the average final concentrations in the sediment and electrolytes. The number of readings was three and, on average, the standard deviations between readings were below 5% both for tests conducted at 70 and 95 days, respectively.

#### 3.3.1 70-day electrokinetic decontamination

**Fig. 4:**
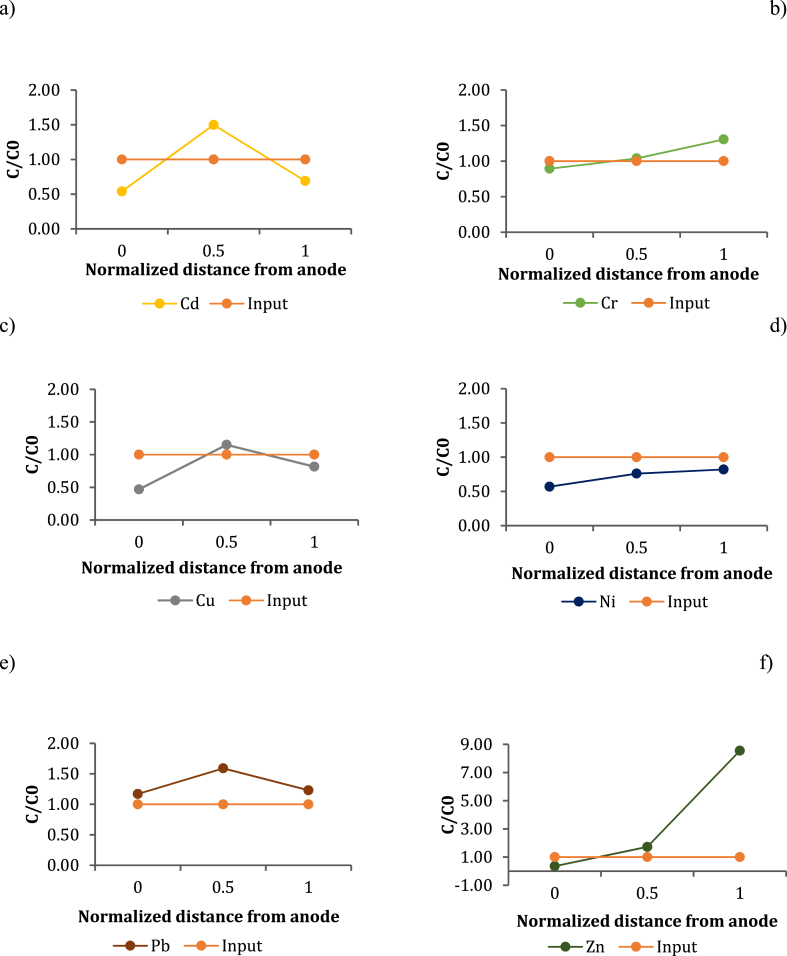
Heavy metal removal at the end of 70 days of EK treatment for Cd (a), Cr (b), Cu (c), Ni (d), Pb (e), Zn (f).

The concentration of heavy metals was monitored at three different points of the portion of sediment treated at an increasing distance from the anode, in order to assess the transport and removal efficiency of contaminants along the matrix. The results obtained after 70 days are shown in Fig. 4 for Cd (a), Cr (b), Cu (c), Ni (d), Pb (e) and Zn (f). The concentration of heavy metals removed was measured on a homogenized fraction of the P84 sample. In general, greater removal efficiency was achieved near the anode. The oxidizing acid front led to the leaching of metals in the part of sediment closest to the anode, involving the transport and accumulation of metals towards the cathode. The combination of multiple factors, such as pH change, migratory ion flow and electroosmotic transport resulted in an efficient decrease in heavy metal concentration in the first section of the curve and, on the other hand, caused a progressive accumulation, as the distance from the anode increased.

In the case of Pb, the concentration reached the maximum value in the middle of the cell, and then decreased approaching the cathode. Interesting, a similar trend was also observed for Cu.

In general, the concentration decreased with respect to input value, suggesting an initial removal of pollutants. Nevertheless, in most cases, the content of metals increased towards the cathode. It was particularly evident for Zn and Cr, where the final concentration increased considerably by exceeding the initial value.

According to other authors, the phenomenon of heavy metal accumulation in some sections of sediments is mainly related to the pH change, which occurs when the process takes place at greater distances from the anode [29]. However, it must also be taken into account that the high inhomogeneity of the sample results in a low accuracy of the measurement of the initial concentration.

The increase in pH by moving away from the acid front favored the precipitation and, consequently, the accumulation of metals such as hydroxides or carbonates.

To better understand the nature of the elements and their precipitation conditions, Masi et al. [1] conducted a speciation study as a function of the pH solution using the Visual MINTEQ calculation model. The results obtained confirmed that most of the metals were in the soluble form at pH < 7. In contrast, Cu and Pb were found to be soluble only at pH < 5, while at pH values ranging between 5 and 7 they may precipitate, depending on the experimental conditions, as CuO, Pb(CO_3_)_2_(OH)_2_ o PbCl(OH), thus explaining the particular trend of some metals [30,31]. In this study, pH was monitored at the anode, cathode, and central section, respectively (Fig. S4). As observed, pH increased from anode to cathode. The acid buffer ability of the sediment inhibited the transport of H^+^ ions, preventing the acid front spreading towards the cathode. As a result, the sections closest to the anode were strongly acidified with a pH value of about 2; on the other hand, moving towards the central section, the pH value increased up to 7, and decreased to 6 in the section next to the cathode. This pH value is not suitable for the solubilization of metals such as Pb, as they precipitate and lead to accumulation in the sediment sections where the pH is close to neutral; nevertheless, the process was particularly efficient for breaking down metals such as Ni and Cu, showing yields greater than 40% after 70 days.

#### 3.3.2 95-day electrokinetic decontamination

**Fig. 5:**
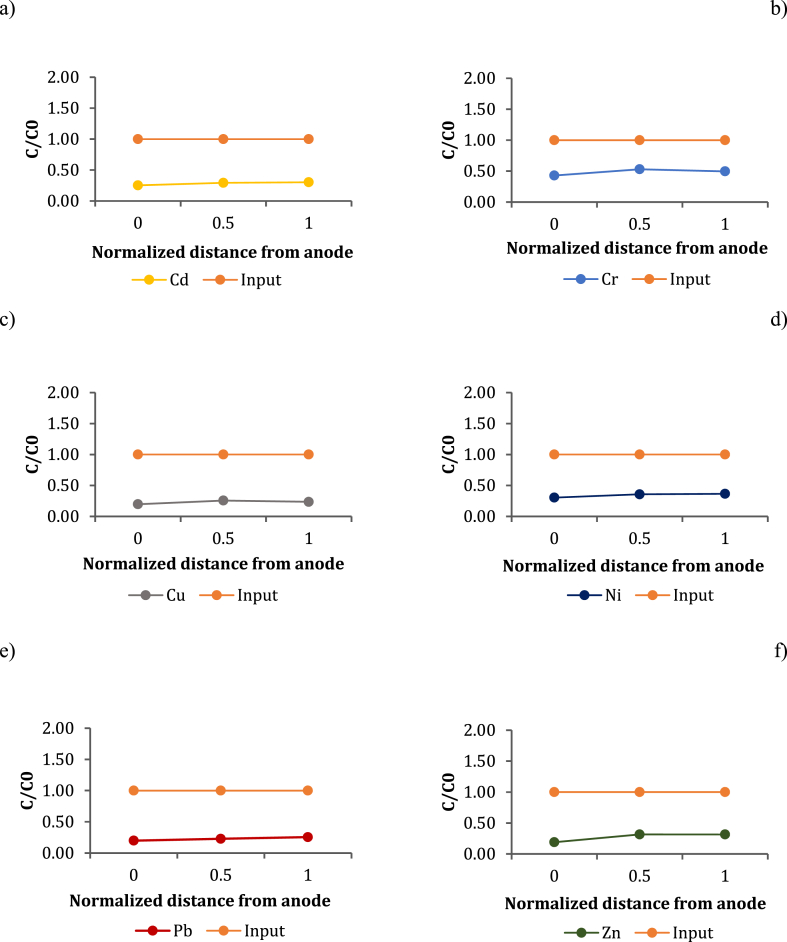
Heavy metal removal at the end of 95 days of EK treatment for Cd (a), Cr (b), Cu (c), Ni (d), Pb (e), Zn (f).

Data obtained after 95 days provided better results, since the removal efficiencies of Cd, Cr, Ni, Pb, Cu e Zn were higher than 50% (Fig. 5a–f). In these cases, metal concentrations at the end of the process were much lower than the initial one, with a slightly higher value in the middle sections and in those closest to the cathode. This trend reflected the pH variation along the sediment. The use of a magnetic drive pump, combined with the lower acid neutralization capacity due to the hexagonal geometry, allowed a complete acidification of the matrix, maintaining pH between 2 and 3 even in the sections farthest from the anode (Fig. S5). In particular, the strong reduction in pH allowed metals such as Pb (which is soluble only at pH < 5) to move into solution, so as to avoid their precipitation and accumulation and facilitate transport and removal mechanisms. As result, by increasing treatment times, it was possible to achieve higher removal rates even for metals that were hardly removed after 70 days, such as Pb, Zn and Cd. Moreover, at the end of 95 days of the EK treatment, an additional amount of around 50% of Pb and Cd were removed, the percentage of removal of Zn, Cr and Cu increased by 30%, while 61.5% of Nickel was removed (a percentage removal of 46.7% was reached after 70 days). In this case it is interesting to note that despite the high content of residual fraction (see Section 3.1), the electrokinetic treatment allowed efficient removal of Nickel both after 70 and 95 days.

#### 3.3.3 Mass balance

**Fig. 6:**
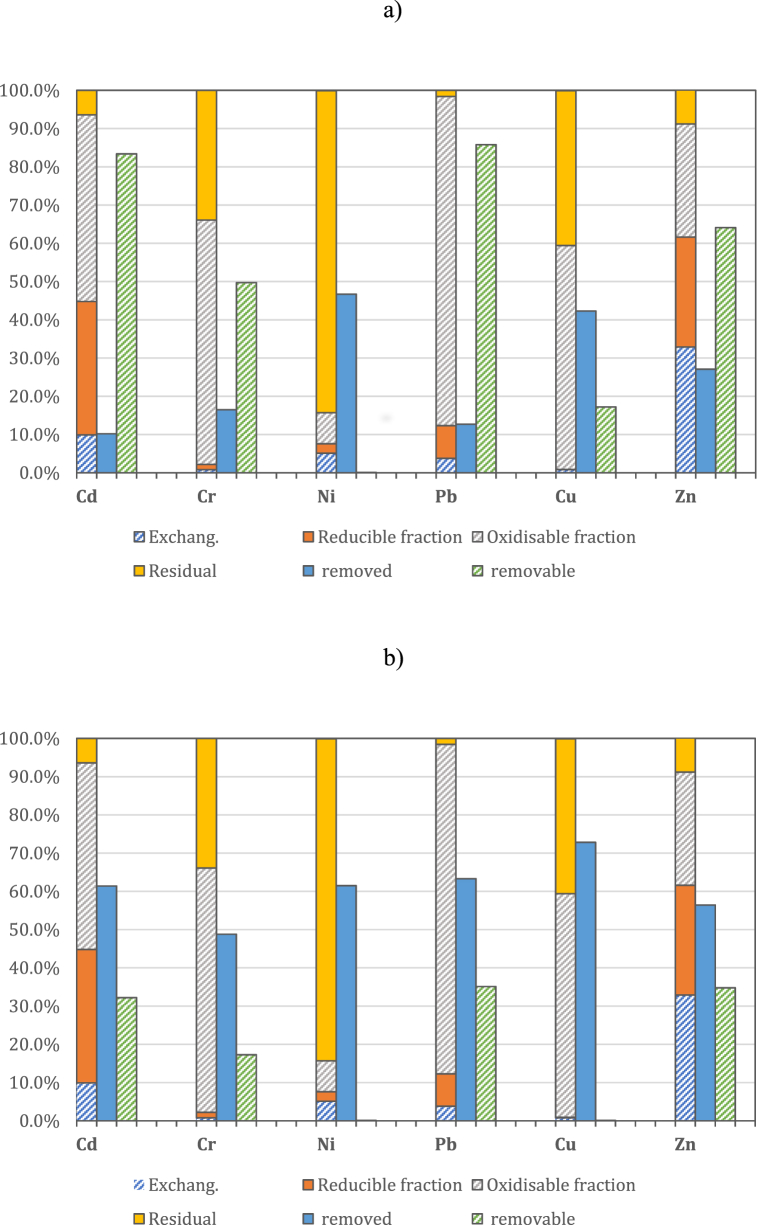
Heavy metal removal comparison with initial speciation at the end of 70 days (a) and 95 days (b), respectively.

To better understand the removal capacity of inorganic contaminants via a hexagonal configuration system, the results obtained at the end of 70 and 95 days were compared with the initial fractionation data (Fig. 6a and b).

Furthermore, the difference between the concentration of heavy metals removed by EK treatment (fraction already removed) and the still bioavailable one that EK technology was unable to remove (removable fraction) was reported.

After 70 days, the unremoved fraction was still higher than the removed ones, especially for Pb, Zn and Cd, which were widely present even at the end of the EK treatment. However, residual samples were strongly linked to the organic matter, which hindered remediation process. In fact, after 70 days of treatment, metal removed content was close to the maximum removable concentration (59%) only for copper (42.3%), while it was less than 30% for all other target metals. In the case of Ni, the residual content was higher than the available concentration, probably due to a measurement error, so it was not considered. The results obtained after 95 days, however, showed higher percentages of removal than the concentration still available, reflecting a successful remediation process. At the end of the 95-day EK treatment, more than 50% of Cd, Pb, Cu and Zn were removed. In addition, the remaining fraction of Cu was negligible, indicating that it was efficiently removed.

### 3.4 Energy consumption

The energy required for dredged marine sediment remediation was computed based on the applied current and the duration time of treatment (70 and 95 days, respectively). The energy consumption per m^3^ of treated sediment was equal to 1547 kWh after 70 days; whereas, after 95 days of experiments, the energy consumption increased to 2705 kWh/m^3^. The results were summarized in Table S3. Compared to similar studies in the literature, the energy required in our study was higher. For instance, the energy consumed by Benamar et al. [13] for the EK treatment of 0.024 m^3^ of marine sediment via linear configuration system was 407 kWh/m^3^. The authors highlighted that, by increasing treatment times (56 days), the energy required per unit volume in a large-scale reactor was more than twice that in a small-scale reactor. However, although it resulted in higher energy consumption, our study provided better performance in terms of heavy metal removal efficiency after only 70 days of treatment (10,17% Cd, 16,46% Cr, 42,29% Cu, 12,65% Pb, 27,08% Zn, respectively) than Benamar et al.’s study (0% Cd, 22,7% Cr, 5,3% Cu, 9,5% Pb, 0% Zn, respectively) [13].

In this context, the environmental impact of EK technology on marine sediment contaminated by heavy metals was evaluated using a Life Cycle Assessment (LCA) by Vocciante et al. [16]; as mentioned by the authors, the consumption of energy represents a critical aspect for global warming potential, accounting for 73% of the total CO_2_ equivalent emissions.

For this reason, the use of renewable energy sources represents a promising alternative, in order to obtain high process yields with a lower impact on the environmental footprint.

### 3.5 Literature comparison

To evaluate the efficiency of a hexagonal configuration in removing heavy metals from dredged marine sediments, since the current study did not employ different EK configuration on the P84 sediment, a comparison with similar studies showing different configurations was reported, based on literature data retrieved over the last ten years. A recent study by Benamar et al. [13] concerned the EK remediation of marine sediments dredged in a Normandy harbor. Large-scale tests (40 kg of sediment) were conducted in a rectangular polyvinyl chloride cell (80 cm × 20 cm × 25 cm), consisting of two electrode compartments (each containing a graphite electrode) and a sediment chamber. A mixture of citric acid and Tween 20 combined with a voltage gradient of 0.5 V/cm were considered optimal conditions for EK decontamination of PAHs/PBCs and metals. The results showed a greater decrease in the concentration of heavy metals in the central sections, especially for Cr and Pb. However, the accumulation of metals in some sections of the reactor hampered their removal (0% Cd, 22,7% Cr, 5,3% Cu, 9,5% Pb, 0% Zn, respectively). Although a shorter treatment time (56 days) was taken into account, the hexagonal configuration system showed significantly higher removal efficiencies, increasing the removal of Cd, Cu, Pb and Zn by around 50–60%. In addition, considering the data obtained over a period closer to 56 days (e.g., 70 days), it is worth noting that the hexagonal configuration system provided better percentage removal, except for Cr (22.7% vs 16.5%), suggesting that the type of configuration should play a key role in the EK processes. Nevertheless, it should be considered that the energy consumption in our study (1547 KWh/m^3^ after 70 days and 2705 KWh/m^3^ after 95 days) was definitely greater compared to the cited work (407 KWh/m^3^ after 56 days).

Another similar study was conducted by Tian et al. [32] to assess the EK treatment of marine sediments, contaminated by heavy metals (Cd, Cr, Cu, Zn and Pb) and PAH/PCB, by using a mixture of eco-friendly agents (rhamnolipids, saponin and citric acid).

The sediments were dredged in a France harbor and placed inside the central chamber of a polytetrafluoroethylene (PTFE) cell (4.9 cm of diameter and 14.2 cm length). During the EK tests, a voltage gradient of 1 V/cm was applied, and the contaminant concentrations were measured at the end of the treatment period (28 days). The results obtained (Table S4) showed a low decontamination efficiency of heavy metals, with a maximum removal of 27.8% of Cr. As mentioned by the authors, a previous study conducted by Ammami et al. [26] allowed to achieve a Zn removal higher than 50%, and generally better performance also for other heavy metals such as Pb and Cd, by adopting different experimental conditions (Table S4). According to other authors [33], it may be related to the high organic matter content in the sediments (11%). Undissolved organic matter can adsorb heavy metals, reducing their mobility. On the other hand, the use of CA as a conditioning agent does not facilitate the desorption process, resulting in poor decontamination. In the case of our study, the organic matter content is quite high, but the use of nitric acid for metal mobilization, combined with longer treatment times and a hexagonal configuration, provided better performance.

On the other hand, the success of an EK process is strictly related to pH conditioning. The effect of different conditioning agents (e.g., H_2_SO_4_, HNO_3_, HCl) in the removal of heavy metals from marine sediments was evaluated by Iannelli et al. [4]. Sediment samples were dredged in an Italian port, and the experiments were performed in a plexiglass cell with sizes of 18 cm (L) × 7 cm (W) × 8 cm (H) at a constant current density of 20 A/m^2^.

Considering the results obtained using HNO_3_, the authors achieved removals of 20.7% of Cr, 16% of Ni, 22.3% of Pb, 17.5% of Cu, 9.5% of Zn after 63 days of treatment. In the case of Ni and Zn, the use of nitric acid promoted electroosmotic flow to the anode, resulting in a lower decontamination rate. However, although it is not recognized as one of the best conditioning agents (such as HCl), the use of HNO_3_ has the advantage of avoiding the development of chlorine gas emissions to be treated.

Furthermore, the use of HNO_3_ showed higher removal efficiencies for metals such as Pb, which, on the contrary, would precipitate as PbSO_4_ if H_2_SO_4_ was used as a conditioning agent instead of HNO_3_ even at low pH. In our study, EK treatment using HNO_3_ resulted in higher removal efficiencies for all of the considered metals, except for Pb after 70-day full test (12.7%). However, longer treatment times (95-day full test) increased the removal of Pb up to 63.3%, meaning that a removal percentage greater than 50 % was reached by the hexagonal configuration process for all the target metals considered.

### 3.6 Limitations

Although our study showed satisfactory results, some limitations occurred: firstly, the high uncertainty on the initial concentration of heavy metals in the sediment due to the high inhomogeneity of the sample; secondly, the use of non-renewable energy sources. In fact, the use of renewable energy sources could allow to investigate the efficiency and functionality of the process in a wider current range. From the authors' point of view, these aspects deserve to be explored further in future research to provide a broader and more accurate knowledge of the process.

## 4 Conclusion

An EK pilot-scale process with hexagonal configuration was applied in this study provided for by the remediation of marine sediments. Preliminary results after 70 days of treatment were reported with an average removal of Cr, Ni, Pb, Cu, Zn of 16.46%, 46.72%, 12.65%, 42.29%, 27.08%, respectively. The duration of treatment and the conditioning of pH are the main factors that affect the process efficiency. Longer treatment times can improve process performance, although the concentration of metals such as Pb and Zn exceeds the limit concentration value provided for by current Italian legislation and the energy consumption increases. Regardless, the hexagonal configuration allowed to remove most heavy metals with high yields (48.80% of Cr, 61.53% of Ni, 63.30% of Pb, 72.84% of Cu, 56.30% of Zn after 95 days of treatment, respectively), especially when compared to other studies on the remediation of marine sediments contaminated by heavy metals with a linear configuration. Therefore, in optimal conditions (pH < 5, applied current density of 30 A/m^2^) hexagonal electrokinetic treatment provided a promising alternative for the removal of heavy metals from dredged sediments even on a large scale.

## Funding

This research was funded by INTERREG ITALIA-FRANCIA MARITTIMO 2014–2020- GRRinPort “Gestione sostenibile dei rifiuti e dei reflui nei porti”, grant number UniCa-Prot. N. 0082843 del 09/05/2018-[Classif. III/19], and the APC was funded by INTERREG ITALIA-FRANCIA MARITTIMO 2014–2020.

## CRediT authorship contribution statement

**Erika Pasciucco:** Writing – original draft, Visualization, Data curation. **Francesco Pasciucco:** Writing – review & editing, Visualization, Investigation, Data curation. **Alessio Castagnoli:** Writing – review & editing, Investigation, Data curation. **Renato Iannelli:** Validation, Supervision, Resources, Methodology, Funding acquisition, Conceptualization. **Isabella Pecorini:** Writing – review & editing, Validation, Supervision, Software, Resources, Project administration, Methodology, Investigation, Funding acquisition, Conceptualization.

## Declaration of competing interest

The authors declare that they have no known competing financial interests or personal relationships that could have appeared to influence the work reported in this paper.
