# Supplementary material for: Removal of heavy metals from dredging marine sediments via electrokinetic hexagonal system: A pilot study in Italy
Source: Heliyon. 2024 Mar 13;10(6):e27616. doi: 10.1016/j.heliyon.2024.e27616 (PMC10955240; doi:10.1016/j.heliyon.2024.e27616)
Supplement: Multimedia component 1 [file mmc1.docx]

**Removal of heavy metals from dredging marine sediments via electrokinetic hexagonal system: a pilot study in Italy**

Erika Pasciucco^a^, Francesco Pasciucco, Alessio Castagnoli, Renato Iannelli, Isabella Pecorini^a*^

^a^Department of Energy, Systems Territory and Construction Engineering, Via C.F. Gabba 22, Tuscany, University of Pisa, Pisa, 56122, Italy

*Corresponding author

**Supplementary materials**

a)


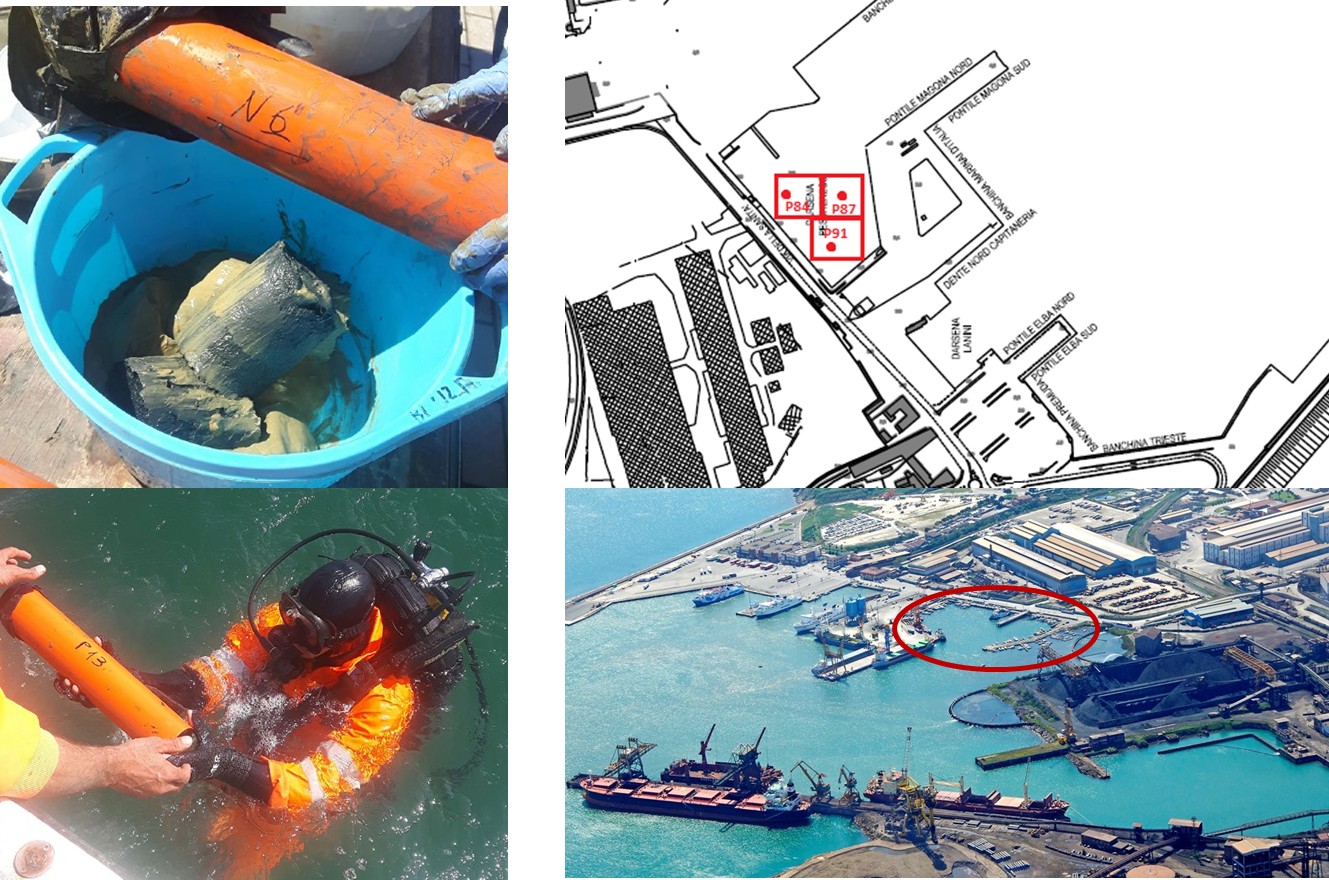


b)

**Figure S1**: Sampling area of P84 dredged sediment (a) and particle size distribution of P84 sample (b).

**Table S1**: experimental conditions of voltage (average value) and current (fixed value) applied during EK tests after 70 and 95 days, respectively.

| Test time (days) | Voltage (V) | Current (A) | Current density (A/m^2^) |
| --- | --- | --- | --- |
| 70 | 7.31 ± 2.18 | 5.18 ± 1.54 | 24.89 ± 14.13 |
| 95 | 8.37 ± 3.73 | 5.80 ± 2.07 | 27.86 ± 16.92 |

a)


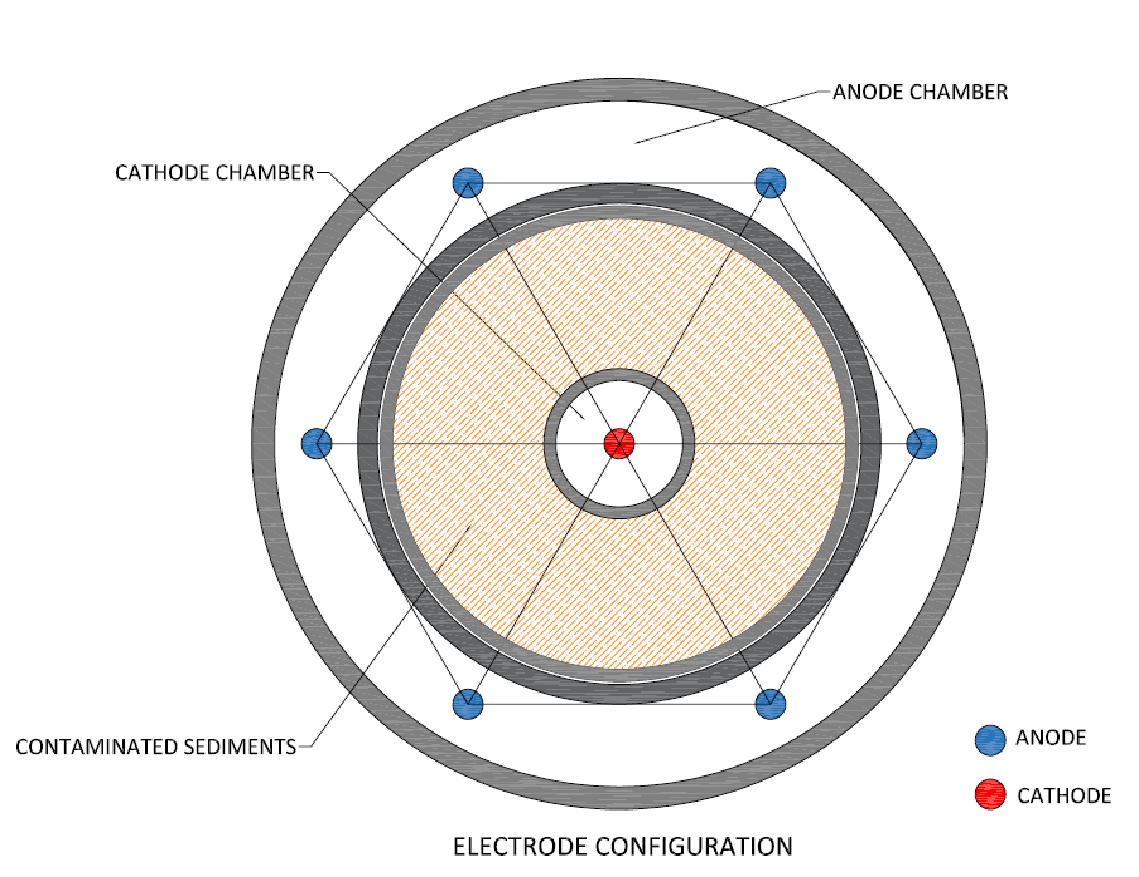


b)


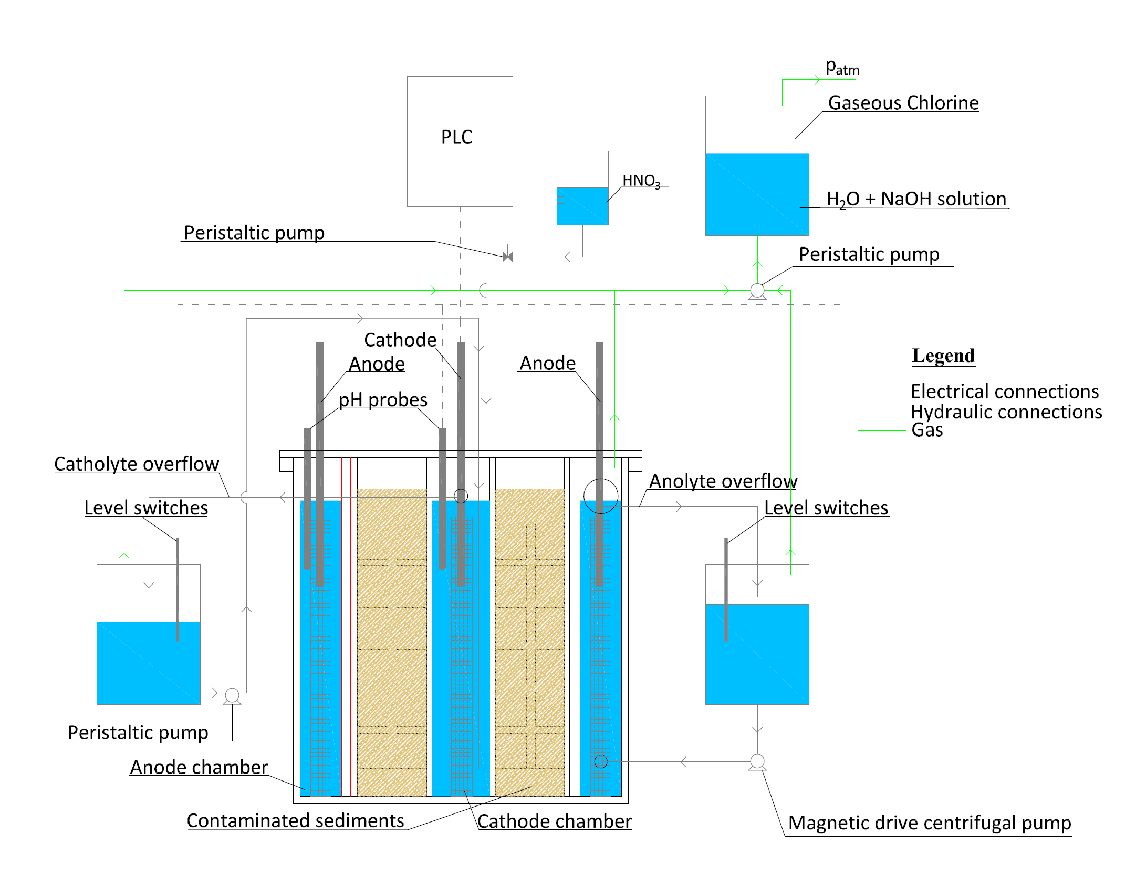


c)


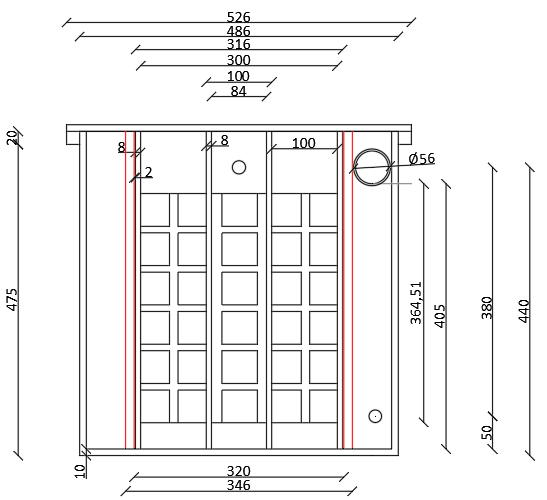


**Figure S2**: schematic representation of the pilot scale system (a), representation of prototype components (b) and their size (c).

**Figure S3**: Voltage trend during EK treatment.

**Table S2**: BCR extraction procedure (Moćko & Wacławek, 2004).

| Step | Extraction procedure | Metal fraction |
| --- | --- | --- |
| 1 | 40 cm^3^ of 0.11 M CH_3_COOH per 1 g dry soil shaken overnight at 25 °C | Exchangeable, water and-acid-soluble species |
| 2 | 40 cm^3^ of 0.1 M NH_2_OH.HCl (adjusted to pH = 2 with HNO_3_) extracted overnight at 25 °C | Reducible |
| 3 | 10 cm^3^ of 8.8 M H_2_O_2_ added to residue, 1-h digestion at 25 °C followed by 1-h digestion at 85 °C. Evaporation, cooling and subsequent extraction of the residue overnight with 50 cm^3^ 1.0 M CH_3_COONH_4_ (adjusted to pH = 2 with HNO_3_), at 25 °C | Oxidisable |

**Figure S4**: pH distribution of sediment during the 70-day tests.

**Figure S5**: pH distribution of sediment during the 95-day

**Table S3**: energy consumption.

| Volume of treated sediment  (m^3^) | Treatment duration (days) | Current density (A/m^2^) | Energy consumption (kWh) | Energy consumption (kWh/m^3^) |
| --- | --- | --- | --- | --- |
| 0,03 | 70 | 30 | 46.61 | 1547 |
| 0,03 | 95 | 30 | 81.15 | 2705 |

**Table S4**: heavy metal removal efficiency (Tian et al., 2017).

|  | Cd | Cr | Cu | Pb | Zn |
| --- | --- | --- | --- | --- | --- |
| EK1 | 9.1 | 17.9 | 5.9 | 2.9 | 3.1 |
| EK2 | 14.4 | 15.8 | 5.7 | 4.4 | 5.8 |
| EK3 | 12.7 | 27.8 | 1.6 | 5.3 | 1.9 |

*EK1: rhamnolipids (1.1 g L^−1^) and citric acid (0.2 mol L^−1^).

*EK2: mixture of saponin (0.85 g L^−1^) and citric acid (0.2 mol L^− 1^).

*EK3: a mixture of saponin (0.85 g L^−1^) and citric acid (0.1 mol L^− 1^).
